# The Immunomodulatory Capacity of Induced Pluripotent Stem Cells in the Post-stroke Environment

**DOI:** 10.3389/fcell.2021.647415

**Published:** 2021-03-16

**Authors:** Samantha E. Spellicy, David C. Hess

**Affiliations:** ^1^MD-Ph.D. Program, Medical College of Georgia at Augusta University, Augusta, GA, United States; ^2^Dean’s Office, Medical College of Georgia at Augusta University, Augusta, GA, United States

**Keywords:** induced pluripotent stem cells, stroke, iNSCs, neuroinflammation, stem cells

## Abstract

Inflammation has proven to be a key contributing factor to the pathogenesis of ischemic and hemorrhagic stroke. This sequential and progressive response, marked by proliferation of resident immune cells and recruitment of peripheral immune populations, results in increased oxidative stress, and neuronal cell death. Therapeutics aimed at quelling various stages of this post-stroke inflammatory response have shown promise recently, one of which being differentiated induced pluripotent stem cells (iPSCs). While direct repopulation of damaged tissues and enhanced neurogenesis are hypothesized to encompass some of the therapeutic potential of iPSCs, recent evidence has demonstrated a substantial paracrine effect on neuroinflammation. Specifically, investigation of iPSCs, iPSC-neural progenitor cells (iPSC-NPCs), and iPSC-neuroepithelial like stem cells (iPSC-lt-NESC) has demonstrated significant immunomodulation of proinflammatory signaling and endogenous inflammatory cell populations, such as microglia. This review aims to examine the mechanisms by which iPSCs mediate neuroinflammation in the post-stroke environment, as well as delineate avenues for further investigation.

## Introduction

Stroke remains a leading cause of death and long-term neurological disability in the aging population worldwide. Despite recent therapeutic efforts and improved control of stroke risk factors, the mean global lifetime risk of stroke has increased over the past 20 years. Historically, this disease has predominantly affected older adults, with 36.6% of individuals over 60 experiencing a stroke (Virani et al., 2020). There is an unmet need for expansion of effective therapeutic options for acute ischemic stroke (AIS).

Current FDA-approved AIS therapeutics are primarily aimed at recanalization of the ischemic vessel through thrombolytic or endovascular means (Powers et al., 2018). While these therapeutic options do restore blood flow to deprived cells in the penumbra (Campbell et al., 2019), they possess no direct mechanisms for neuroprotection or neuronal salvage Additionally, they come with an increased risk of intracranial hemorrhage (Meinel et al., 2020), hemorrhagic transformation (Yaghi et al., 2017), and 50% of thrombectomy patients remain disabled at 3 months post-stroke (Goyal et al., 2016). For these reasons, there is an overwhelming need for the investigation, trial, and implementation of novel therapeutic options for stroke sufferers.

Inflammation has proven to be a key contributing factor to the pathogenesis of ischemic and hemorrhagic stroke (Buscemi et al., 2019; Jayaraj et al., 2019), representing an avenue for novel AIS therapeutic targets (Iadecola and Anrather, 2011). The post-stroke neuroinflammatory environment is marked by a multi-stage progressive sequence of resident immune cell activation, peripheral immune cell recruitment, and the release of inflammatory cytokines and reactive oxygen species (ROS). In the initiation phase, endothelial cells, perivascular macrophages, platelets, and neutrophils are responsible for a release of proinflammatory cytokines (IL1β, IL-1α, and TNF-α), chemokines (CCL5, CXCL4, CXCL7), and proteases (MMP8, MMP9, MT6-MMP) (Iadecola and Anrather, 2011; Jayaraj et al., 2019). In turn, newly activated microglia release pro-inflammatory cytokines, such as interleukin (IL)-1β, IL-6, and tumor necrosis factor (TNF)-α, resulting in the amplification phase—activating and recruiting nearby astrocytes, dendritic cells, and T-cells (Iadecola and Anrather, 2011). Infiltrating T-lymphocytes, which peak around 3 days post-AIS, are responsible for perpetuating apoptosis and blood-brain barrier (BBB) breakdown through continued cytokine secretion (Yoshimura and Shichita, 2012). This cascade can lead to astrocyte activation up to 28 days after stroke (Dabrowska et al., 2019). This acute and subacute post-stroke proinflammatory cascade presents a multi-faceted avenue for anti-inflammatory therapeutic options, with an array of potential therapeutic targets and an increased temporal window compared to current thrombolytic and endovascular approaches. Specifically, iPSCs and iPSC-derived cells have exhibited significant immunomodulation of endogenous inflammatory cell populations and proinflammatory signaling.

### Induced Pluripotent Stem Cells as a Stroke Therapeutic

**FIGURE 1:**
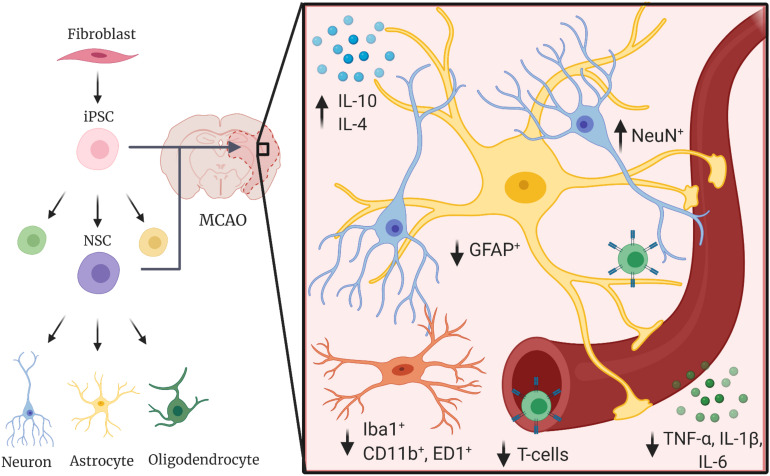
Induced pluripotent stem cells decrease proinflammatory responses in the post-stroke environment. iPSCs and iPSC-derived cells have been shown to decrease proinflammatory cytokines such as TNF-α, IL-1β, and IL-6, decrease activation and polarization of local immune cells such as astrocytes (GFAP+) and microglia (Iba1^+^, CD11b^+^, and ED1^+^), and increase anti-inflammatory cytokines (IL-10, IL-4) in animal models of ischemic and hemorrhagic stroke.

iPSCs are a viable regenerative therapeutic in a range of degenerative conditions. These cells, which can be induced from somatic cells to pluripotent stem cells (Takahashi and Yamanaka, 2006; Warren et al., 2010; Li et al., 2014), allow new opportunities for generation of neural stem cells and neural cell lineages with less ethical concerns than embryonically derived stem cells (Zacharias et al., 2011; Buzanska, 2018). The limitless potential for self-renewal, proposed lower rate of transplantation rejection of autologous cells than embryonic stem cells (De Rham and Villard, 2014), and ability for genetic engineering, make them an attractive therapeutic option (Suda et al., 2020). Here, the anti-inflammatory potential of iPSCs and differentiated lineages in models of stroke is further discussed and outlined in Figure 1.

### Non-differentiated iPSCs

#### iPSCs as a Therapy in Ischemic Models of Stroke

Rodent middle cerebral artery occlusion (MCAO) models of stroke have served as a means in which to test the anti-inflammatory potential of iPSCs. While some paradigms utilize a permanent occlusion, others invoke transient occlusions ranging from 30- to 90- min (Shahjouei et al., 2016). This occlusion, often through filamentous blockade of the MCA, induces ischemic damage to the ipsilateral striatum and dorsolateral cortex (Chiang et al., 2011). Paradigm specifics for the studies included in this review are detailed in Table 1. In one such model, iPSC and fibrin glue treatment resulted in a significant decrease of interleukin (IL)-1β, TNF-α, IL-2, and IL-6 and iNOS expression in the lesion cortex compared to non-treated groups. Additionally, protein levels of IL-4 and IL-10 were significantly elevated in the iPSC- and fibrin-treated group compared to MCAO-only animals (Chen et al., 2010). This study reveals iPSCs have the capacity to decrease inflammatory reactivity and polarization of innate immune cells as well as attenuate circulating proinflammatory mediators in the local ischemic stroke environment. Future studies should be aimed at assessing if measured anti-inflammatory cytokines, such as IL-4 and IL-10, are secreted from transplanted and differentiated iPSCs or if the iPSCs cause an indirect attenuation of proinflammatory responses through direct action on innate immune modulators.

**TABLE 1:** Studies assessing the efficacy and anti-inflammatory potential of iPSCs in models of ischemic and hemorrhagic stroke.

Cell-type	Model	Inflammatory finding	Mechanism of administration	Functional findings
Mouse primary iPSCs (WP5 line) (Chau et al., 2014)	Permanent MCAO in P7 Wistar rat pups	↑ Expression of SDF-1 in iPSC stroked animals compared to sham animals	Direct injection of ∼1 × 10^5^ (4 sites) into peri-infarct region 7 days post-stroke	No significant difference in left paw reaches when compared to sham animals
Rat iPSCs (Qin et al., 2015)	Collagenase induced intracerebral hemorrhage (ICH) stroke in Sprague-Dawley rat model	↓Protein and RNA levels of IL-1β, IL-6, and TNF-α ↑ Expression of IL-10 2 days post-ICH. ↓ MPO^+^ and CD11b^+^ cells as well as a decreased expression of activated caspase-3^+^/NeuN^+^ cells 3 days post ICH. ↓Damage to Nissl bodies ↓GFAP^+^ expression in ISC groups at day 42 post-ICH	Direct injection of 1 × 10^6^ cell (3.5 mm deep relative to the bregma, 2.5 mm upon the hemorrhagic lesion) into the parenchyma, 6 h post-ICH	↑ Modified limb placing test score compared in iPSC compared to vehicle treated
C57/B6 mouse MEF derived (13.5 day old embryos) iPSCs (Chen et al., 2010)	Proximal tMCAO (1 h) in Long-Evans rats	↓ Il-1b, TNF-α, Il-2, and Il-6 in iPSC + fibrin glue ↓ Expression of iNOS, ↑ IL-4 and IL-10 in iPSC + fibrin glue	Direct subdural injection 1 h post-MCAO, immediately after ischemic reversal	↓ Infarct volume in iPSC + fibrin glue ↑ Latency to fall on rotarod and increased grasping power of left forepaw in iPSC + fibrin glue compared to control at 1, 2, 3, and 4 weeks post MCAO
Human iPSC-NSCs (551-8 hPSC line) (Chang et al., 2013a)	Transient MCAO (90 min) Sprague-Dawley (SD) rats	↓IBA1^+^, round ED1^+^, and GFAP^+^ cells in the ischemic core 8 weeks post-transplant	Direct injection of 1 × 10^5^ cells/mL into the contralateral side of the infarct regions (P + 1.0 mm, ML + 3.0 mm, and DV −5 mm from the Bregma) 7 days post-MCAO	–
Human (20 year-old male) iPSC-NSCs (Lee et al., 2017)	Permanent right MCAO and transient bilateral common carotid occlusion (1 h) in Long-Evans rats	−↓ED1^+^ cells No GFAP-immunoreactive astrogliosis ↓ TUNEL^+^ neurons and GFAP^+^ astrocytes in OGD injury ↓ CXCL14	1 × 10^6^ cells in epidural fibrin glue over the infarct cortex	↑ Grip strength, ↓ Ipsilateral forearm use bias, and infarct volume
Human (CD34^+^ cord donor blood) iPSC-NSCs (Basuodan et al., 2018)	Endothelin-1 injection in sensorimotor cortex at P12 in Wistar rats	↓Reactive microglia found at the boundary of the graft if iPSC-NSC cells compared to EMC-alone grafts 4 weeks post grafting	Directly transplanted intracerebrally (in ECM) into the ischemic Sensorimotor cortex at day 14 post-stroke	–
Murine iPSC-NSCs (Yamashita et al., 2017)	Transient MCAO (35 min) in C57BL/6N mice	↓ GFAP^+^ expression in the cortex and striatum of iNSC treated animals at 7 days post-stroke, but not at 28 days post-stroke No significant difference in IBA1^+^ expression was observed at either time	Direct injection into the ipsilateral striatum and cortex (Anterior −0.5, 2.5 mm lateral, and 1.5–2.5 mm deep) 24 h after ischemia induction	↑ survival, Bederson’s score, and corner test No significant difference in infarct volume or rotarod at day 7 post MCAO
Human (viral transgene -free and IMR90-1, IMR90 clone 1) iPSC-NSCs (Eckert et al., 2015)	Transient MCAO (60 min) in C57BL/6J mice	↓ Active IBA^+^ cells, TNF-α, IL-6, IL-1β, ICAM-1, VCAM-1, MCP-1, MIP-α of iNSCl, IgG, and MMP9 treated animals compared to non-treated animals. ↑ BDNF	Direct injection of ∼1 × 10^5^ cells into the ipsilesional hemisphere (2 mm Posterior, 1.5 mm Lateral, and 2–2.5 mm Dorsal to the bregma) 24 h post-MCAO/R	↓ Time for adhesive removal and beam walk ↑ Rotarod time
Human (HIP^TM^ hNSC BC1) iPSC-NSCs (Baker et al., 2017)	Permanent MCAO in landrace pigs	↓IBA1^+^ cells in iPSC-NSC treated vs. non-treated animals No change in GFAP^+^ cells from non-treated animals	Direct injection into the infarcted parenchyma 5 days post-MCAO	–
Human iPSC-lt-NESCs (Adult male dermal fibroblasts) (Tatarishvili et al., 2014)	Permanent distal MCAO and 30 min bilateral carotid occlusion in Sprague-Dawley rats	No differences in the number of IBA^+^ microglia/macrophages ↓Number of activated ameboid IBA^+^ cells in treated animals. Weak VEGF staining	Direct injection of ∼3 × 10^5^ cells in 2 injection sites in the injured cerebral cortex (1.5 mm anterior, 1.5 mm lateral, and 2–2.5 mm deep to the bregma) 28 h post-dMCAO	↑ Performance on left paw touches on cylinder test in treated group. No significant improvements over control in left backhand, right backhand, left forehand, and right forehand adjustments on stepping test pattern following stroke compared to non-stroked

**Results of studies which examined anti- or pro-inflammatory markers and responses of iPSC or iPSC -derived cells in models of stroke. Functional results of treated animals are also included if assessed in the study.**

In contrast to this ischemic stroke study, other studies suggest a less defined role of iPSCs in the stroke environment. One rodent dMCAO model of stroke found a significantly increased expression of SDF-1/CXCL12 (stromal derived factor-1/lymphocyte, C-X-C motif ligand 12) in the peri-infarct area of iPSC-treated animals above that of non-stroked animals. No significant difference, however, was observed in the expression of SDF-1 between stroked non-treated animals and iPSC-treated animals (Chau et al., 2014). While SDF-1/CXCL12 is a stroke chemotactic signal for lymphocytes, in the central nervous system it serves to recruit endogenous NSCs to areas of injury for tissue repair (Li et al., 2012). Therefore, the increased observed expression of this chemokine following iPSC treatment could be a sign of increased neuroregeneration in these ischemic areas. Further investigation into the predominating effect of increased CXCL12 in the post-stroke environment following iPSC treatment is warranted to assess the comprehensive effect on the inflammatory balance.

#### iPSCs as a Therapy in Hemorrhagic Models of Stroke

Outside of ischemic models of stroke, investigation of the anti-inflammatory potential of iPSCs has been conducted in intracranial hemorrhagic (ICH) (Théry et al., 2018) models of stroke. In a collagenase-induced hemorrhagic rat stroke model, significantly decreased protein and RNA levels of IL-1β, IL-6, and TNF-α, and significantly increased expression of IL-10, were observed at 2 days post-ICH in iPSC-treated animals compared to PBS controls.

Immunohistological quantification on day 3 post-ICH also revealed a significant decrease in MPO^+^ (myeloperoxidase, granulocytes, and monocytes) and CD11b^+^ (cluster of differentiation 11b, microglia/macrophages) cells, as well as a decreased expression of activated Caspase-3^+^ (cellular apoptosis)/NeuN^+^ (neuronal nuclei, neurons) cells. Lastly, Nissl (nucleic acids) staining at day 42 post-ICH revealed less damage to Nissl bodies and nuclei within neural cells in the iPSC-treated group than the PBS (vehicle) group, and significantly decreased GFAP^+^ expression at day 42 post-ICH was detected in iPSC-treated tissue (Qin et al., 2015). This study demonstrates the anti-inflammatory mechanisms of iPSCs are not limited to the ischemic stroke environment but are observed in rodent models of the hemorrhagic stroke, as well.

#### Limitations

While there is compelling evidence of the positive effect of non-differentiated iPSCs on the stroke inflammatory environment, there are limitations associated with this proposed therapeutic. In the non-stroke condition, mouse models have revealed an increased tumorigenicity of iPSCs over iPSC-NSCs, NSCs, and MSCs similar to that of ESCs (Gao et al., 2016). Models of the stroke condition, specifically, have found similar results regarding iPSCs tumorgenicity (Yamashita et al., 2011). In a direct comparison of iPSCs and iPSC-NSCs in a MCAO rat model of stroke, iPSCs were found to have an increased tumorigenic potential, while iPSC-NSCs were not (Kawai et al., 2010; Wu et al., 2015). Due to the proposed increase in safety, further consideration of the therapeutic potential of iPSC-NSCs should be discussed.

### Induced Pluripotent Neural Stem Cells (iPSC-NSC)

Through the use of specific culture conditions, such as including stromal cocultures (Chang et al., 2013b), the presence or absence of supplemented factors, and the selection of nestin-positive cells (Chen et al., 2010; Chang et al., 2013b), iPSCs can be differentiated into a neural lineage. These further-differentiated cells have been a focus of regenerative stroke research, as they are thought to differentiate and repopulate niches of damaged neuronal and glial cells after injury (Oki et al., 2012; Zhang et al., 2012; Chang et al.,2013a,b; Tornero et al., 2013; Sugai et al., 2016). Of note, attenuation of reactive gliosis in the stroked animal brain does not impact neural stem cell engraftment and neurogenesis (Laterza et al., 2018), revealing iPSCs-NSCs to be a robust therapeutic option in the harsh stroke neuroinflammatory environment. Additionally, these cells are believed to secrete neuroprotective and regenerative factors which promote increased recovery and improved functional outcomes (Jiang et al., 2011; Polentes et al., 2012; Chang et al., 2013b; Boese et al., 2018). Recent focus has been directed on the anti-inflammatory mechanisms of these secreted factors and their potential to dampen the post-stroke neuroinflammatory environment through a “bystander effect” (Smith and Gavins, 2012).

#### *In vitro* Assays of iPSC-NSCs

To further delineate potential paracrine effects of iPSC-NSCs, an *in vitro* oxygen glucose deprivation (OGD) model was conducted in which iPSC-NSCs were arranged in a membrane-separated co-culture system with OGD cortical cells (Lee et al., 2017). In this model, there was a significant decrease in TUNEL^+^ (deoxynucleotidyl transferase dUTP nick end labeling) and GFAP^+^ cells as well as significant increases in residual neurons and neurites of iPSC-NSC groups compared to OGD cells alone. Interestingly, this pattern of responses was also seen in iPSC groups but was not observed in bone marrow-derived mesenchymal stem cells (BM-MSCs) or Wharton’s jelly MSC groups. Therefore, these antiapoptotic and anti-inflammatory responses seems to be specific to iPSCs and iPSC-NSCs. Messenger RNA expression level analysis revealed a significant increase in BMP7 (bone morphogenic protein 7), CXCL14 (C-X-C motif 14, chemoattractant for monocytes and dendritic cells), FGF12 (fibroblast growth factor 12, regulation of voltage-gated Na^+^ channels), and FGF8 (neuronal migration) over iPSCs and MSCs, as well as FGF9 (radial glia migration), and IGFBP2 (insulin-like growth factor protein) over MSCs. While most of these factors have known implications in neuronal development and NSC differentiation, factors such as BMP7, CXCL14, and IGFBP2 have been known to play a role in anti- and proinflammatory processes as well (Lee et al., 2017).

Cytokine array analysis of culture supernatants also revealed a significant enrichment of IGFBP2 in iPSC-NSCs over BM-MSCs and OGD-injured cultures. When these cytokines (BMP7, CXCL14, FGF8, FGF9, and IGFBP2) were individually dosed onto OGD cells, a significant neuroprotective and anti-inflammatory phenotype was observed upon immunocytochemical quantification—similar to that observed with iPSC-NSC co-culture. Specificity of these factors to carry out mechanisms of neuroprotection and anti-inflammation was confirmed by a neutralizing antibody cocktail which abolished these effects (Lee et al., 2017). Lastly, the entire rat transcriptome in OGD-injured cortical cells co-cultured with iPSC-NSCs or BM-MSCs was analyzed and only 26 over expressed genes were common between OGD and IPSC-NSCs or BM-MSC co-cultures. Specifically, iPSC-NSCs had significantly increased expression of genes involved in neurogenesis (Notch1, epidermal growth factor receptor, CXCL12) and anti-apoptosis (Lee et al., 2017). Collectively, these *in vitro* experiments demonstrate that iPSCs-NSCs not only exert anti-inflammatory, neurogenic, and anti-apoptotic effects on stressed neuronal cultures, but also that these effects are distinct from other stem cell sources (e.g., MSCs).

Extracellular matrices (ECM) can also enhance the anti-inflammatory potential of iPSC-NSCs. In non-stroke *in vivo* conditions, grafted iPSC-NSCs in ECM form rosettes, develop radial glia, and are vascularized by 8 weeks post-engraftment. Interestingly, while cell-free ECM grafts were found to have reactive microglia accumulating at the boundary of the graft, only quiescent host microglia were found within the organoid structures of the iPSC-NSC + ECM group 4 weeks post-engraftment. These results suggest iPSC-NSCs promote an anti-inflammatory environment *in vivo*, which is not contingent on an ischemic or hemorrhagic condition, and that they protect against chronic innate immune cell activation following trauma (Basuodan et al., 2018).

#### *In vivo* Assays of iPSC-NSCs

Gene expression data in *in vivo* rodent MCAO models revealed significant decreases in TNF-α, IL-6, IL-1β, intercellular adhesion molecule 1 (ICAM-1), vascular cells adhesion molecule (VCAM-1), monocyte chemoattractant protein (MCP-1), and macrophage inflammatory protein (MIP-α) expression of iPSC-NSC-treated animals compared to non-treated animals. Additional protein level analysis revealed a significant decrease in mouse immunoglobulin G (IgG) and matrix metallopeptidase 9 (MMP9) (Eckert et al., 2015). MMP9 is believed to be released by circulating neutrophils after they have adhered to damaged cerebrovascular endothelium to decrease BBB integrity and aid in neutrophil extravasation. This migration of neutrophils into the damaged cerebral space is thought to induce more ROS and MMP9 production from endogenous immune cells (e.g., microglia), resulting in increased neuroinflammation and neural cell death (Turner and Sharp, 2016). The accompanying decreased levels of IgG in this study suggests increased BBB integrity, hinting that an anti-inflammatory effect on neutrophils by iPSC-NSCs may be responsible for the decreased MMP9 (Eckert et al., 2015). More work is needed to ascertain if the decreased levels are also due in part to decreased microglial secretion.

Immunocytochemistry data of *in vivo* MCAO models of ischemic stroke have also found similar anti-inflammatory and neuroregenerative potential of iPSC-NSCs. Studies in rodent models have found significant reductions in IBA1^+^ (Chang et al., 2013a; Eckert et al., 2015), ED1^+^ (Chang et al., 2013a; Heo et al., 2013; Lee et al., 2017), and GFAP^+^ cells (Chang et al., 2013a; Lee et al., 2017) in the ischemic core of iPSC-NSC-treated animals compared to non-treated animals. Additionally, the number of apoptotic cells post-MCAO measured through TUNEL assay and the number of activated inflammatory cells has been shown to be significantly reduced in iPSC-NSC treated animals (Chang et al., 2013a; Lee et al., 2017) as far as 8 weeks post-transplantation. In contrast, while a transient MCAO mouse model of stroke revealed a significant decrease in GFAP expression in the cortex and striatum of iPSC-NSC-treated animals at 7 days post-stroke, a difference was not seen at 28 days post-stroke. Additionally, no significant difference in IBA1 expression was observed at either time point in this study (Yamashita et al., 2017). Interestingly, these animals did have improved survival, corner test results, and Bederson score. These differences in the specific anti-inflammatory results may be due to the timing of administered iPSC-NSC to animals following MCAO. While the majority of studies which found significant reductions in IBA^+^ and GFAP^+^ cells in the acute and chronic stroke environment administered the therapeutic at subacute time points (∼7 days post-MCAO), the latter study administered cells at an acute (24 h) timepoint. This difference could have implications for the therapeutic window of iPSC-NSCs to exert their effects.

To assess the translational potential of iPSC-NSCs as a clinical stroke therapeutic, their anti-inflammatory efficacy in large animal models of stroke must also be considered. The increased complexity, size, and cellular organization of large animal models serve to assess scaling, dosing, or diffusion-limited processes. Additionally, due to a white-to-gray matter composition which more closely resembles humans, differential effects of iPSC-NSCs on these cells can be assessed (Ann-Christine et al., 2003; Kuluz et al., 2007; Ahmad et al., 2015; Kaiser and West, 2020). In a porcine MCAO model of stroke, there was a significant reduction in IBA1^+^ area in iPSC-NSC-treated animals compared to non-treated animals. There was not a significant reduction in GFAP^+^ cells however (Baker et al., 2019). While this study serves as preliminary evidence of the anti-inflammatory effects of iPSC-NSCs in more complex systems, further studies in large animal models are needed.

Similar to iPSC-NSCs, neuroepithelial-like stem cells have also been differentiated from iPSCs and are distinct from radial glia-like NSCs (Falk et al., 2012). The efficacy of these cells has also been assessed in a range of ischemic stroke paradigms. In a dMCAO model of stroke, iPSC-lt-NESCs were transplanted into the cerebral cortex of rats 48 h post-stroke. There was a significantly decreased number of ameboid activated microglia in transplanted groups compared to vehicle treated animals (Tatarishvili et al., 2014). Further investigation into the secreted factors necessary to govern this cell fate distinction could give a glimpse into the post-stroke inflammatory milieu following iPSC-lt-NESC treatment.

### Future Directions

Outside of the lineages discussed in this review, there are many iPSC-derived lineages which exhibit noted anti-inflammatory effects but have not yet been investigated in the context of stroke. For example, iPSCs-MSCs (Eto et al., 2018; Zhao and Ikeya, 2018) have been shown to be safe and invoke anti-inflammatory responses in models of irritable bowel syndrome (Yang et al., 2019), graft vs. host disease (Ozay et al., 2019; Bloor et al., 2020), and periodontitis (Yang et al., 2014). Additionally, BM-MSCs have demonstrated anti-inflammatory mechanisms in ischemic and hemorrhagic models of stroke (Boshuizen and Steinberg, 2018; Li et al., 2018; Turnbull et al., 2019), such as decreasing ED1 + expression (Heo et al., 2013). While it is recognized that BM-MSCs have reduced differentiation potential in extended culture (Jung et al., 2012), iPSCs-MSCs have not demonstrated this limitation. Therefore, based on the known anti-inflammatory effects of iPSC-MSCs in systemic models of disease, and the documented efficacy of BM-MSCs in stroke, iPSC-MSCs could prove a viable therapeutic alternative to BM-MSCs.

While the functional effects of iPSCs and iPSC-derived cells in stroke are clear—decreasing lesion volume (Chen et al., 2010; Lee et al., 2017) and mortality (Yamashita et al., 2017) and improving functional recovery (Chen et al., 2010; Tornero et al., 2013; Tatarishvili et al., 2014; Eckert et al., 2015; Qin et al., 2015; Lee et al., 2017; Yamashita et al., 2017)—the precise timing, sequence, and degree to which anti-inflammatory mechanisms play in these observed functional effects has yet to be fully elucidated. In addition, recent reports have pointed to a biphasic role of post-stroke inflammatory processes with beneficial and detrimental effects (Jayaraj et al., 2019). Determination of the temporal components of these iPSC anti-inflammatory mechanisms will be essential for optimal dosing of iPSCs as well as artfully preserving the acute beneficial post-stroke proinflammatory responses. In regard to therapeutic administration, distinguishing if anti-inflammatory mechanisms are carried out in a paracrine or contact-mediated manner can help determine if transplanted cells must be successfully engrafted to carry out their anti-inflammatory activity. Lastly, tumorigenicity of iPSC cells, a product of *in vitro* culture expansion and reprogramming, has remained a major hurdle to clinical implementation. This complex discussion involves consideration of the implications of single nucleotide variants, mutations in non-coding regions, and mutations in recognized cancer genes. Whether these mutations would lead to cancerous proliferation or affect the therapeutic potential of iPSC cells remains a topic of active debate (Yamanaka, 2020) and warrants consideration of standardized tumorigenicity tests (Kuroda et al., 2013) and inhibitors of teratoma formation (Gorecka et al., 2019). Assessment of these properties will be essential in ascertaining the clinical translational potential of iPSCs as a human stroke therapeutic.

## Author Contributions

SS and DH designed, wrote, and edited the manuscript and have approved it for publication. Both authors contributed to the article and approved the submitted version.

## Conflict of Interest

The authors declare that the research was conducted in the absence of any commercial or financial relationships that could be construed as a potential conflict of interest.
